# Mitochondrial role in adaptive response to stress conditions in preeclampsia

**DOI:** 10.1038/srep32410

**Published:** 2016-08-30

**Authors:** Polina A. Vishnyakova, Maria A. Volodina, Nadezhda V. Tarasova, Maria V. Marey, Daria V. Tsvirkun, Olga V. Vavina, Zulfiya S. Khodzhaeva, Natalya E. Kan, Ramkumar Menon, Mikhail Yu. Vysokikh, Gennady T. Sukhikh

**Affiliations:** 1 Research Center for Obstetrics, Gynecology and Perinatology, Ministry of Healthcare of the Russian Federation, 4, Oparina street, Moscow, 117513, Russia; 2 Belozerskii Institute of Physico-chemical Biology, Moscow State University, Moscow, Leninskie gory 1, 119992, Russia; 3 Department of Obstetrics and Gynecology, University of Texas Medical Branch at Galveston, TX, 77555, USA

## Abstract

Preeclampsia (PE) is a pregnancy-specific syndrome, characterized in general by hypertension with proteinuria or other systemic disturbances. PE is the major cause of maternal and fetal morbidity and mortality worldwide. However, the etiology of PE still remains unclear. Our study involved 38 patients: 14 with uncomplicated pregnancy; 13 with early-onset PE (eoPE); and 11 with late-onset PE (loPE). We characterized the immunophenotype of cells isolated from the placenta and all biopsy samples were stained positive for Cytokeratin 7, SOX2, Nestin, Vimentin, and CD44. We obtained a significant increase in OPA1 mRNA and protein expression in the eoPE placentas. Moreover, TFAM expression was down-regulated in comparison to the control (p < 0.01). Mitochondrial DNA copy number in eoPE placentas was significantly higher than in samples from normal pregnancies. We observed an increase of maximum coupled state 3 respiration rate in mitochondria isolated from the placenta in the presence of complex I substrates in the eoPE group and an increase of P/O ratio, citrate synthase activity and decrease of Ca^2+^-induced depolarization rate in both PE groups. Our results suggest an essential role of mitochondrial activity changes in an adaptive response to the development of PE.

Preeclampsia (PE) is a pregnancy-specific syndrome, characterized by hypertension with proteinuria or thrombocytopenia, renal insufficiency, impaired liver function, cerebral or optical disorders or pulmonary edema after the 20th week of gestation^1^,^2^. PE affects 2–8% of all pregnancies worldwide and still remains the major cause of maternal and fetal death^2^,^3^. This disease is characterized by a decrease of trophoblast invasion and abnormal remodeling of spiral arteries^4^. Specialists distinguish two types of PE: early-onset and late-onset, depending on gestation age. Most investigators consider early-onset PE (eoPE) as that occurring before 34 weeks and late-onset PE (loPE) occurs after this time^5^,^6^. EoPE is typically characterized by intrauterine growth restriction, decrease of placental weight, low baby mass, perinatal death, and unfavorable outcomes. LoPE is marked by normal placenta weight, normal fetal growth, normal baby weight, and more favorable outcomes^7^. Despite the large body of data, PE etiology remains unclear. However, it is now known that placental insufficiency plays a key role in the progression of this disease.

PE was first suggested to be a mitochondrial disorder at the end of 1980s^8^. Later, it was shown that mitochondrial dysfunction in the PE placenta induces oxidative stress^9^,^10^,^11^. There is accumulating evidence for antioxidant system decline (down-regulation of superoxide dismutase and glutathione peroxidase) and up-rise of reactive oxygen species (ROS), mainly produced by mitochondria at PE^12^,^13^,^14^. Loss of mitochondrial control of ROS levels in the cell makes a significant contribution to the pathophysiology of PE. Ultrastructural data, obtained with electron microscopy, showed mitochondrial swelling and vacuolation, along with the disappearance of cristae in mitochondria from trophoblast cells of PE placentas^15^,^16^. Taken together, partial functional incompetence and altered morphology of mitochondria reflect common features of mitochondrial disorders presented in PE. Both morphology and function strongly depend upon the state of mitochondrial biogenesis, including fission, fusion and mitochondrial DNA (mtDNA) turnover, transcription and respiration rate. However, the number of studies that have investigated placental mitochondrial molecular machinery during PE is limited. Thus, the aim of our study was to identify mitochondrial structural and functional properties in placenta samples from three groups of pregnant women: eoPE, loPE, and normal pregnancies. In our work, we examined the bioenergetics of placental mitochondria, the expression level of factors that play a key role in mitochondrial fusion (mitofusin-1–MFN1, mitofusin-2–MFN2, mitochondrial dynamin like GTPase – OPA1), fission (dynamin-related protein 1–DRP1), biogenesis (nuclear respiratory factor 1–NRF1), mitochondrial permeability (voltage-dependent anion-selective channel protein 1–VDAC1) and activation of mtDNA transcription (mitochondrial transcription factor A–TFAM).

## Results

Clinical characteristics of all women who took part in the study are shown in Table 1.

### Cell immunophenotype and viability

To characterize cell viability and the cellular composition of placenta, we used flow cytometry analysis. Cells were analyzed after short-term cultivation at first passage and there were no significant differences in percentage of viability (%) between groups: CTRL 73.4 ± 3.5; eoPE 76.4 ± 2.9; loPE 68.8 ± 3.1, values shown as mean ± SD. To characterize cell population structure we chose five proteins: trophoblast-specific intracellular marker cytokeratin 7 (Cyt7)^17^, a transcription factor of tropho-ectoderm development SOX2, a marker of endothelial precursors–Nestin and also Vimentin and CD44^18^, markers of mesenchymal and stromal cells, respectively. In all cases, cells from short-term cultivated placental primary cultures were positive for the chosen markers, but there were no significant differences in the percentage of positive-stained cells between groups (Supplementary Table S1).

### Changes in mRNA level

**Figure 1:**
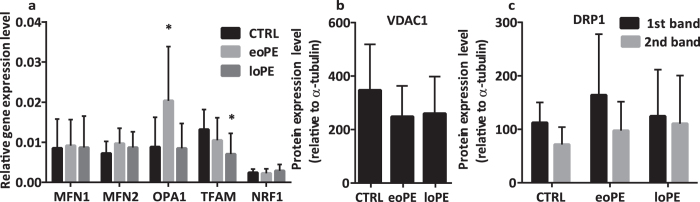
Comparison of mRNA and protein levels in control and PE groups. Relative expression levels of the genes, *MFN1, MFN2, OPA1, TFAM* and *NRF1,* normalized to β-actin (**a**). Relative protein expression level of VDAC1 (**b**) and DRP1 (**c**), normalized to α-tubulin. Values shown are mean ± SD. *p < 0.01 versus control. n_(CTRL)_ = 14, n_(eoPE)_ = 13, n_(loPE)_ = 11.

Mitochondrial fusion and fission play a key role in mitochondrial quality control and are controlled by the nuclear genome. We chose three genes essential for mitochondrial fusion (*MFN1, MFN2, OPA1*), biogenesis (*NRF1*) and the *TFAM* gene, which is an activator of mtDNA transcription. To determine whether there is a difference between mRNA expression among groups, we used quantitative RT-PCR, using the β-actin gene as internal reference (Fig. 1a). We observed a significant 2.5-fold increase of *OPA1* relative expression level in the eoPE group in comparison with control placenta samples (p = 0.001), whereas there was no difference between control and loPE. Simultaneously, no changes were found either in *MFN1, MFN2* or *NRF1* relative expression levels for all investigated groups. However, we found that the *TFAM* level was 1.8-fold lower in loPE placentas (p = 0.005), while, in the eoPE group, expression had no difference compared to the control group.

### Protein expression of VDAC1, OPA1, DRP1, and TFAM in placentas from preeclamptic and normal pregnancies

**Figure 2:**
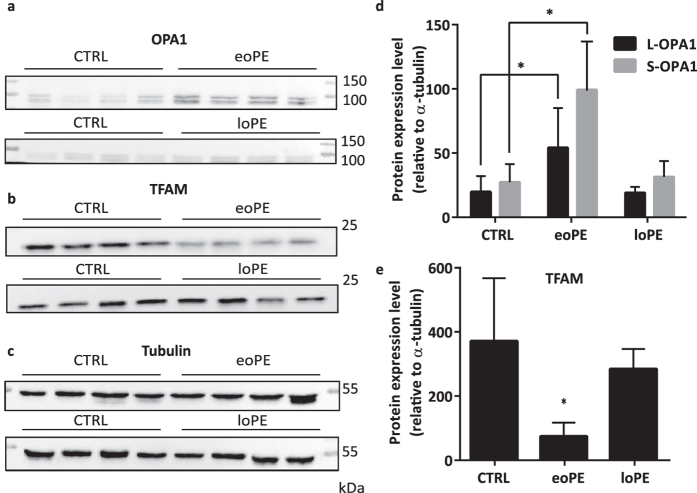
Expression of studied proteins in placental tissue. Anti-OPA1 (**a**), anti-TFAM (**b**), anti-tubulin (**c**) staining of placental homogenates. Relative protein expression level of OPA1 (**d**) and TFAM (**e**) normalized on α-tubulin. Values shown are mean ± SD. *p < 0.01 versus control. n_(CTRL)_ = 14, n_(eoPE)_ = 13, n_(loPE)_ = 11.

Protein expression level of VDAC1, a major protein found in the outer mitochondrial membrane, appeared to be the same in both preeclamptic and control placentas (Fig. 1b). For OPA1, an inner membrane fusion protein, we compared the level of two forms: cleaved (S-OPA1) and uncleaved (L-OPA1). We found that relative expression of both OPA1 forms was 3-fold higher (p < 0.0001 for S-OPA1 and p = 0.0004 for L-OPA1) in eoPE placentas than in normal ones (Fig. 2a,d). On the other hand, OPA1 level did not change in loPE compared to control. Interestingly, there was no difference in DRP1 expression level, an important protein responsible for mitochondrial fission (Fig. 1c). We obtained a 5-fold decrease of TFAM expression in eoPE group in comparison with normal pregnancies (p = 0.002). As Fig. 2b,e shows, a similar effect was observed in loPE samples, but this result was not significant (p > 0.05). Anti-OPA1 and anti-TFAM staining of all remaining samples from studied groups is available in Supplementary Information (Supplementary Fig. S1).

### Immunohistochemistry of placenta

**Figure 3:**
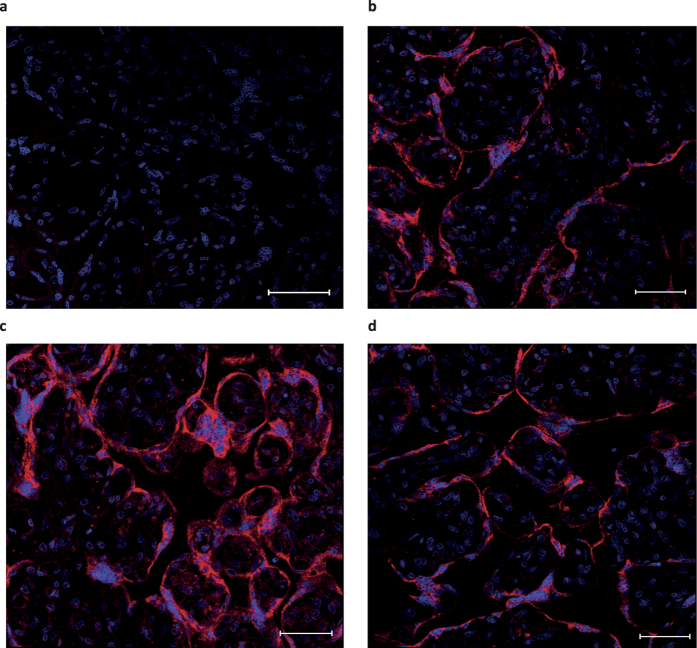
Immunohistochemical staining for OPA1 in control, eoPE and loPE placental tissues. Isotype control (**a**). Normal (**b**), eoPE (**c**) and loPE (**d**) placentas were stained with anti-OPA1 (red) antibody (40-fold magnification). Nuclei were visualized by DAPI (blue). Bars mean 50 μm.

To verify OPA1 protein level changes we performed pilot immunohistochemical experiment with 3 placenta samples from each studied group. Representative images are shown in Fig. 3. Staining for OPA1 was 1.5-fold more intense in the placental tissue from eoPE compared with the normal placentas (Mean fluorescence intensity (MFI): isotype-matched control 15.0 ± 0.5; CTRL 33.3 ± 3.6; eoPE 48.2 ± 3.2*; loPE 33.8 ± 5.8, values shown as mean ± SD, *p < 0.01 versus control) and this result is consistent with Western blotting analysis. Staining in loPE was similar to control placentas.

### Determination of mtDNA quantity and citrate synthase activity

**Figure 4:**
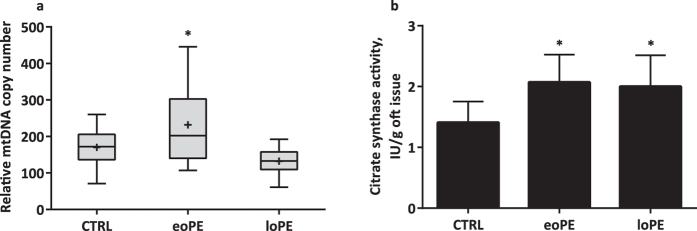
Distribution of relative mtDNA copy number and citrate synthase activity of placenta samples in control, eoPE and loPE groups. The mtDNA content (**a**) was measured by RT-qPCR normalizing the quantity of a not-polymorphic region of D-loop and MT-ND2 gene with a single copy nuclear gene (β-2-microglobulin). The median (line), mean (cross) and 25–75% interquartile range (IQR) are shown. n_(CTRL)_ = 14, n_(eoPE)_ = 13, n_(loPE)_ = 11. Citrate synthase activity assay (**b**) was performed on placenta homogenates in studied groups. n_(CTRL)_ = 5, n_(eoPE)_ = 5, n_(loPE)_ = 5. *p < 0.05 versus control.

mtDNA copy number was measured in frozen placenta samples by qPCR using primers for the D-loop region, the MT-ND2 gene, and the single copy nuclear gene–β-2-microglobulin. We observed a significant 1.5-fold increase (p = 0.04) of relative mtDNA copy number in the eoPE group. mtDNA copy number in control and loPE placentas were not significantly different from each other (Fig. 4a). To verify if increase in mtDNA content in eoPE is associated with increase in mitochondrial mass, we performed citrate synthase activity assay. We observed significant increase in citrate synthase activity in both PE groups (p < 0.05) (Fig. 4b).

### Mitochondrial respiration

**Figure 5:**
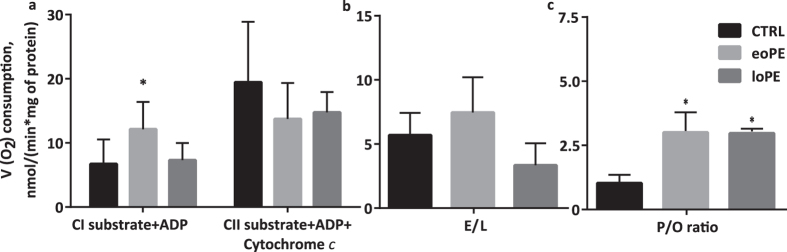
Respiration of placental mitochondria. Maximum rate of coupled respiration in presence of complex I (CI) and– complex II (CII) substrates (**a**). E/L is the ratio of respiratory electron transfer system capacity (E) of mitochondria in the experimentally induced noncoupled state to the leak proton flux (L) in presence of complex II substrate (**b**). P/O ratio (phosphate/oxygen ratio) signifies the amount of ATP produced per oxygen atom reduced by the respiratory chain (**c**). Values shown are mean ± SD. *p < 0.05 versus control. n_(CTRL)_ = 12, n_(eoPE)_ = 8, n_(loPE)_ = 3.

To estimate mitochondrial respiration, we used standard protocols, described in Methods section. The maximum coupled state 3 respiration rate in presence of complex I (CI) substrates was significantly increased in eoPE (p = 0.006 ), but not in loPE compared with the control group (CTRL 6.7 ± 3.8; eoPE 12.3 ± 4.2; loPE 7.3 ± 2.7) (Fig. 5a). The maximum coupled respiration rate in presence of complex II (CII) substrates slightly decreased in both PE groups, but the difference was not statistically significant. We also obtained a tendency to increase the ratio of maximum noncoupled respiration rate to coupled non-stimulated respiration rate (E/L ratio) in presence of CII substrate in the eoPE group (p = 0.07) and a tendency to decrease in the loPE group (p = 0.06) compared to control (CTRL 5.7 ± 1.7; eoPE 7.5 ± 2.7; loPE 3.4 ± 1.7) (Fig. 5b). Moreover, we determined the P/O ratio (phosphate/oxygen ratio)–the number of phosphate atoms incorporated as ATP per atom of oxygen consumed during oxidative phosphorylation. In the presence of CII substrate, the P/O ratio was increased both in eoPE and loPE groups (p = 0.002 and 0.04, respectively) compared to the control group (CTRL 1.1 ± 0.65; eoPE 3.0 ± 1.3; loPE 2.9 ± 0.3) (Fig. 5c).

### Mitochondrial membrane potential

**Figure 6:**
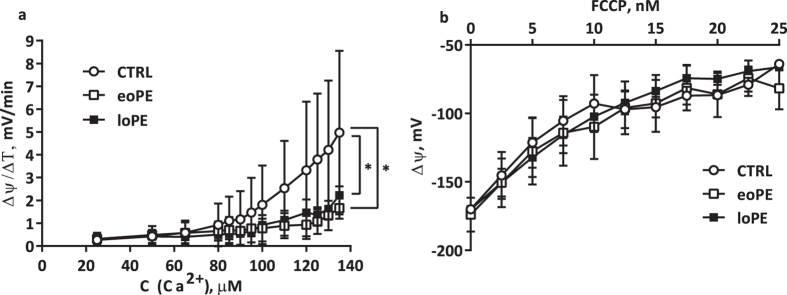
Mitochondrial membrane potential response to titration by Ca^2+^ and FCCP. Rate of Ca^2+^-induced depolarization was determined in mitochondrial suspensions from control and preeclamptic placentas (**a**). Effects of FCCP on *ΔΨ* in mitochondrial suspension from all studied groups (**b**). Values shown are mean ± SD. *p < 0.05 versus control. n_(CTRL)_ = 12, n_(eoPE)_ = 8, n_(loPE)_ = 4.

We measured changes in mitochondrial transmembrane potential caused by application of Ca^2+^ and carbonyl cyanide p-trifluoro-methoxyphenyl hydrazone (FCCP) to a mitochondrial suspension, followed by detection of fluorescence changes of safranin O. As shown in Fig. 6a, we found that calcium sensitivity in both PE groups was significantly lower than in control placentas (p < 0.05). However, we did not observe any significant difference in mitochondrial membrane potential (*ΔΨ*) during FCCP titration (Fig. 6b).

## Discussion

Preeclampsia is a pregnancy-specific syndrome, characterized in general by hypertension with proteinuria or other systemic disturbances. An increase of oxidative stress in the preeclamptic placenta could be explained by a sequential chain of events including poor trophoblast invasion, failed spiral artery remodeling, and an ischemia-like state with further reperfusion caused by fluctuations of oxygen levels in conditions of reduced fetomaternal blood flow^19^. In our study, we estimated the condition of molecular machinery associated with fulfillment of mitochondrial function in placentas from pregnancies complicated with PE.

An important aspect of our study was the characterization of placental biopsy primary cultures after short-term cultivation. There were no significant differences in the cell composition of placentas between the control, eoPE, and loPE groups, although all biopsy samples were stained positive for Cyt7, SOX2, Nestin, Vimentin, and CD44. Despite the result was not substantial we explain such a big difference in percent of Cyt7-positive cells between control and eoPE groups by early gestational age of placenta^20^. Taking together this data could mean that a reason of PE symptoms is not the changes in cellular composition, but rather alterations of cell functioning.

Mitochondrial activity correlates with organelle state and depends on the degree of mitochondrial fragmentation, biogenesis and dynamic of mtDNA turnover rate and stability. The mitochondrial network is highly dynamic and is precisely regulated, especially in stressful conditions. Decreased activity of the antioxidant system and increased ROS could lead to induction of mitochondrial permeability transition (MPT) and, as a result, to the swelling of mitochondria, outer membrane rupture, and proapoptotic factors release^21^. It is well known that calcium homeostasis contributes to the proper functionality of mitochondria–low Ca^2+^ activates matrix dehydrogenases and respiration whereas high concentration of Ca^2+^ promotes MPT^22^. Surprisingly, we found a significant decrease of Ca^2+^-induced mitochondrial membrane depolarization rate in both PE groups in comparison to the control. This decrease of mitochondrial sensitivity to Ca^2+^ could be an adaptive response with aim to prevent excessive apoptosis in placenta caused by MPT opening, manifested in oxidative stress^23^, typical for PE. A recent study by Haché and colleagues^24^ also demonstrated a decrease in calcium transport in mitochondria from preeclamptic syncytiotrophoblasts. On the other hand, we did not find a difference in the rate of *ΔΨ* changes between groups when protonophore FCCP was added to mitochondria.

The index of oxidative phosphorylation efficiency (P/O ratio) increased in both PE groups compared to control. It is known that high P/O corresponds to high value of proton motive force^25^,^26^. However, correlation between proton motive force and mitochondrial membrane potential exists and this fact together with our observation led us to presume probable increased risk of ROS formation by PE placental mitochondria at high *ΔΨ* in state 4^27^. In our case, it is manifested in high efficiency of energy production. Indeed, it is well known that oxidative stress appears in the PE placenta^11^,^14^,^28^,^29^. The high P/O ratio and ROS production seem to be positively coregulated^27^,^30^.

It should be noted, that results concerning mitochondrial bioenergetics were observed on isolated mitochondria derived mainly from placental cytotrophoblast, whereas the remaining results were obtained on placental tissue homogenate. In our study we made an assumption that we observed the same molecular changes in particular mitochondrial fraction and whole tissue homogenate.

Furthermore, it is essential to recognize type and estimate the value of adaptive response of the mitochondria quality control system to PE oxidative stress conditions. Among other proteins, OPA1 is an essential component of the mitochondrial quality control system^31^. This nuclear encoded polypeptide is localized in the inner mitochondrial membrane and takes part in mitochondrial fusion processes. While mutations in the *OPA1* gene induce autosomal dominant optic atrophy (ADOA), this protein also regulates apoptosis and takes part in mtDNA maintenance^32^. Müller-Rischart and colleagues^33^ demonstrated that the *OPA1* gene is a target of NF-κB-responsive promoter elements (e.g. NEMO–NF-κB essential modulator) which is upregulated in stressful conditions, particularly in PE^34^. We observed that in the eoPE group, OPA1 expression was significantly higher compared to control, for both transcript and protein levels. Studies on mice showed that OPA1 overexpression protects mitochondria from apoptotic-related cristae remodeling and cytochrome *c* release events^35^. Thus, in cases of severe forms of pathology, up-regulation of OPA1 in eoPE could be an essential part of the protective mechanism with its role in stabilization of appropriate mitochondrial structures.

Having analyzed mtDNA, we observed increase in copy number in the eoPE group. In maternal blood, mtDNA copy number was also significantly higher in samples from women with PE^36^. Simultaneous up-regulation of OPA1 and an increase in mtDNA copy number could be interrelated in eoPE. Previous works indicate that mtDNA copy number and exon 4b abundance in *OPA1* transcripts are coregulated: exon 4b-encoded peptide could bind to mtDNA to make it available for replication or transcription^37^. Perhaps OPA1 up-regulation promotes increase of mtDNA copy number by stabilization of mitochondrial nucleoid. In addition, a decrease in mtDNA copy number in blood lymphocytes of ADOA-1 patients^38^ and in HeLa cells with OPA1 knockdown^39^ was shown. We hypothesize that OPA1-driven segregation and distribution of mitochondrial nucleoids between mitoplasts occurred before fission and selection of mitochondria/mtDNA are triggered by the mitochondrial quality control system.

We observed an acceleration in coupled respiration on CI (state 3), which was in agreement with increased mtDNA copy number and probability of OPA1-driven cristae remodeling. Kushnareva and colleagues^39^ showed that OPA1 knockdown leads to a reduction of mtDNA copy number, deterioration of mitochondrial Ca^2+^ retention capacity and ADP-induced respiration (state 3) in HeLa cells. Our findings concerning the effect of Ca^2+^ on placental mitochondria, increase in mtDNA copy number and changes in mitochondrial bioenergetics in eoPE are consistent with observations of Kushnareva and colleagues.

Since TFAM is an essential activator of mtDNA transcription, replication and participant of mtDNA packaging^40^,^41^, changes in TFAM expression could generally affect mitochondrial functionality and production of subunits encoded in mtDNA^42^. In the present study, we observed a significant decrease of TFAM mRNA expression in the loPE group and a reduction of TFAM protein in eoPE samples, as compared to the control group. It was widely believed that TFAM expression and mtDNA copy number are co-regulated. Ekstrand and colleagues^43^ observed that TFAM knockout in mouse embryos caused a reduction in mtDNA copy number. In contrast, one study did not reveal the influence of TFAM expression on mtDNA copy number in cells^44^. To explain the increase of mtDNA copy number and low TFAM level in the eoPE group, we used the model of mtDNA titration by TFAM, proposed in review by Kang^45^. According to this model, bound TFAM is involved in architecturally maintaining mtDNA whereas mtDNA-free TFAM is unstable in mitochondria and degrades rapidly. Due to the active replication of OPA1-stabilized mtDNA, TFAM is dissociated and degraded. Then, increased phosphorylating potential and efficiency of mitochondrial respiration in eoPE are required to support the sufficient rate of ATP-dependent LONP1-driven degradation of TFAM^46^ and to ensure functioning of HSP70, up-regulated in PE^12^.

In summary, we proposed that mitochondrial state changes in early-onset preeclamptic placentas are accompanied by OPA1 up-regulation, decrease of Ca^2+^-induced depolarization rate, active mtDNA replication and, as a consequence, a high respiration rate in presence of complex I substrate, high P/O ratio and down-regulation of TFAM. Mechanisms that lead to similar mitochondrial activity changes (Ca^2+^-induced depolarization rate, citrate synthase activity and P/O ratio) in loPE could be different and associated with other molecular pathways. For example, improvement of substrate phosphorylation in mitochondrial matrix based on the mitochondrial phosphoenolpyruvate carboxykinase and the succinate-CoA ligase activity could take place. This process is not dependent from proton motive force and not coupled to respiration, but leads to similar effect in ADP consumption and production of ATP or GTP at conditions of hypoxia^47^. Indeed, this could be indirect explanation of decrease in Ca^2+^-induced depolarization rate due to mitochondrial dehydrogenases are Ca^2+^-dependent enzymes. Last, observed increase of citrate synthase activity allows to suggest activation of substrate phosphorylation in loPE. These aspects are thoroughly investigated and will be described in our future work.

Abnormal trophoblast invasion in the early stages of development results in perturbed gas exchange between the mother, placenta, and fetus. Placental mitochondria begin to work more effectively to compensate for reduced oxygen delivery by enhancing resistance to endogenous uncouplers. Increased calcium sequestering by mitochondria could lead to a decrease of NO production by nitric oxide synthase, which results in inhibition of vasculogenesis and further deterioration of feto-placental blood turnover. Thus, probably there are two processes that run simultaneously–the first is cristae remodeling and the second is the distribution of nucleoids. Both processes are required to ensure the quality of mitochondria. We assume that the probable segregation of mtDNA could precede mitoplast fragmentation, mitochondrial fission, and disruption of the whole mitochondrial reticulum. Such segregation occurs before mitoplast separation underneath the intact outer mitochondrial membrane and could be a prerequisite for further selection of mitochondria with intact mtDNA by the quality control system.

## Methods

### Ethics Statement

All experiments involving placental tissue were conducted in accordance with the Declaration of Helsinki, guidelines for Good Clinical Practice and Commission of Biomedical Ethics at Research Center for Obstetrics, Gynecology and Perinatology, Ministry of Healthcare of the Russian Federation. All experimental protocols were approved by the Commission of Biomedical Ethics at Research Center for Obstetrics, Gynecology and Perinatology. All the patients signed informed consent in accordance with the Ethics Committee requirements and Helsinki Declaration of the World Medical Association.

### Sample collection

Placental tissues were sampled immediately after delivery *via* elective caesarean section (CS) proposed on clinical grounds from women with normal pregnancies, women with eoPE, and loPE in Research Center for Obstetrics, Gynecology and Perinatology in Moscow. Main indications for elective CS in the control group were uterine scar after previous CS, myomectomy and high myopia according to ophthalmologist conclusions. The central area of placental chorionic tissue was dissected, and the maternal decidua and amniotic membranes were removed. Samples were collected from the same area each time (1.5–2 cm next to the umbilical cord insertion, 1 cm in depth) for reducing the bias caused by differences in gene expression within the same placenta depending on sampling site^48^. After being washed in phosphate buffered saline (PBS), the tissue fragments were immediately placed in DMEM/F12 medium (PanEco, Russia) or frozen in liquid nitrogen and stored until use. PE and severity of PE were estimated according to common medical criteria^1^,^49^.

### Cell culture

Primary cultures were obtained from the fetal part of placental villous tissue by enzymatic treatment. Full information could be found in Supplementary Information.

### Cell immunophenotype

The cell immunophenotype was determined by flow cytometry with FACSCalibur (Becton Dickinson, USA) using monoclonal antibodies to Cyt7 (CBL194F, clone LP5K, Millipore, Germany), SOX2 (IC2018P, Clone 245610, R&D Systems, USA), Nestin (FCMAB313PE, clone 10C2, Millipore, Germany), Vimentin (ab128507, clone RV202, Abcam, USA), CD44 (555478, Clone G44-26, BD Pharmingen, USA), labeled with fluorescein isothiocyanate (FITC) or phycoerythrin. IgG of the corresponding class were used as isotype control antibodies (all – BD Pharmingen, USA). 10,000 cells were analyzed in each measurement.

### Immunohistochemistry

Immunohistochemistry was performed according to Abcam IHC staining protocol for paraffin, frozen and free floating sections. Additional information is provided in Supplementary Information.

### RNA extraction and reverse transcription reaction

Samples of placental tissues were homogenized in liquid nitrogen. The powder was dissolved in 1 ml of Extract RNA Reagent (Evrogen, Russia). All procedures were carried out according to the manufacturer’s protocol. RNA concentration and 260/280 ratio was measured with spectrophotometer DS-11 (DeNovix, USA). For the reverse transcription reaction, 0.5 μg of total RNA was reverse transcribed using MMLV-RT kits (Evrogen, Russia).

### Real-Time Quantitative RT-PCR

Quantification of mRNA was performed using a DT-96 thermocycler (DNA-Technology LLC, Russia). Real-time PCR reactions were conducted in a reaction volume of 10 μl, containing 100 ng of cDNA, 300 nM of each primer and 2 μl of 5xSybrGreen-mix (Evrogen, Russia) in triplicate. All primer sequences (Supplementary Table S2) were generated and verified for specificity by Primer-BLAST. 1.5% agarose gel electrophoresis and melting curve analysis were used for amplicon size estimation and primer specificity. The PCR program consisted of an initial step at 95 °C for 5 min, followed by 45 cycles of denaturation at 95 °C for 10 s, annealing at 60 °C for 20 s and elongation at 67 °C for 20 s, followed by melting at a gradient from 65 °C to 95 °C. Relative gene expression was determined as the ratio of the target gene to the internal reference gene expression (β-actin) based on Ct values using QGENE software.

### mtDNA content measurement

Total DNA extraction from placental tissue was performed using Nucleic Acid Extract kit (DNA-Technology, Russia) and precisely quantified with a spectrophotometer, DS-11. The mtDNA content was measured by Real Time PCR, normalizing the quantity of a non-polymorphic region of D-loop and mtDNA-encoded ND2 gene with a single copy nuclear gene (β-2-microglobulin). 100 ng of total DNA were analyzed in triplicate with the same PCR conditions as mentioned above. Relative quantification values were calculated by the 2^−*Δ*Ct^ method^50^.

### Activity of citrate synthase

Citrate synthase activity was determined in placental tissue homogenate as described^51^ at a wavelength of 412 nm.

### Western blot analysis

Western blots were carried out with standard protocol. Full experimental procedure is available in Supplementary Information.

### Mitochondria isolation

Mitochondria were isolated by the method of differential centrifugation. A detailed protocol is located in Supplementary Information.

### Measurement of mitochondria activity

We used the polarographic/amperometric technique to assess respiratory chain complexes efficiency. Full protocols are found in Supplementary Information.

### Determination of mitochondrial membrane potential and sensitivity of mitochondria to Ca^2+^ exposure

Mitochondrial *ΔΨ* measurement was performed in mitochondrial suspension through fluorescence changes of lipophilic cationic dye safranin O. A detailed protocol could be found in Supplementary Information.

### Statistical analysis

Data are presented as mean ± standard deviation (SD) as well as median and 25–75% IQR. The Shapiro-Wilk normality test was used to estimate distribution. One-way analysis of variance (ANOVA) followed by the Tukey’s post-hoc test was used to examine differences among multiple groups with normal distribution. One-way Kruskal-Wallis non-parametric ANOVA followed by the post-hoc Dunn test was used to calculate statistical differences for non-normal distributions. All calculations were performed in STATISTICA 6.0 software (StatSoft, USA), R programming language (The R Foundation) and Prism v6.0 software (GraphPad, USA). P-value < 0.05 was considered as significant and was indicative of the differences in comparison to control.

## Additional Information

**How to cite this article**: Vishnyakova, P. A. *et al*. Mitochondrial role in adaptive response to stress conditions in preeclampsia. *Sci. Rep.* **6**, 32410; doi: 10.1038/srep32410 (2016).

## Supplementary Material

Supplementary Information

## Figures and Tables

**Table 1:** Clinical characteristics of patients.

Characteristics	Control	eoPE	loPE
Number	14	13	11
Maternal age, years	31.5 ± 4.3	34 ± 4.6	29 ± 4.2
Gestational age at delivery, weeks	39.2 ± 0.9	30.5 ± 2.9*	37.8 ± 1.0
Body mass index before delivery, kg/m^2^	26.7 ± 2.3	29.2 ± 4.9	29.8 ± 4.7
Systolic blood pressure, mm Hg	115.0 ± 5.8	161.0 ± 15.5*	149.0 ± 7.7*
Diastolic blood pressure, mm Hg	73.0 ± 5.0	101.0 ± 9.5*	96.0 ± 7.0*
Proteinuria, mg/dL	n.d.	2080.3 ± 1393.1*	1312.3 ± 1440.2*
Sex of the baby (Male/Female), %	50/50	30/70	36/64
Intrauterine growth restriction, %	n.d.	61*	27*
Severe form of PE, %	n.d.	69*	9*
Baby mass, g	3560.7 ± 405.6	1295.3 ± 586.2*	2828.5 ± 494.2*

Data are listed as mean ± SD.

^*^р < 0.01 versus control; n.d.–not detected.
