# Supplementary material for: Mitochondrial role in adaptive response to stress conditions in preeclampsia
Source: Sci Rep. 2016 Aug 30;6:32410. doi: 10.1038/srep32410 (PMC5004102; doi:10.1038/srep32410)
Supplement: Supplementary Information [file srep32410-s1.pdf]

SUPPLEMENTARY INFORMATION

Mitochondrial role in adaptive response to stress conditions in preeclampsia

Polina A. Vishnyakova, Maria A. Volodina, Nadezhda V. Tarasova, Maria V. Marey, Daria V. Tsvirkun, Olga V. Vavina, Zulfiya S. Khodzhaeva, Natalya E. Kan, Ramkumar Menon, Mikhail Yu. Vysokikh and Gennady T. Sukhikh.

Supplementary Table S1. Heterogeneity of the cells from placental primary culture

	% of positive-stained cells				
	Cyt7	SOX2	Nestin	Vimentin	CD44
CTRL	31.8±18.7	27.2±9.2	58.0±13.5	14.0±3.5	77.25±9.2
eoPE	65.6±19.5	31.0±23.7	72.0±6.6	16.5±7.5	59.5±10.5
loPE	18.75±8.9	11.0±6.0	62.6±13.4	11.0±2.5	88.0±9.0

Data is presented as mean ± SEM. $n_{(CTRL)}=11$, $n_{(eoPE)}=5$, $n_{(loPE)}=4$

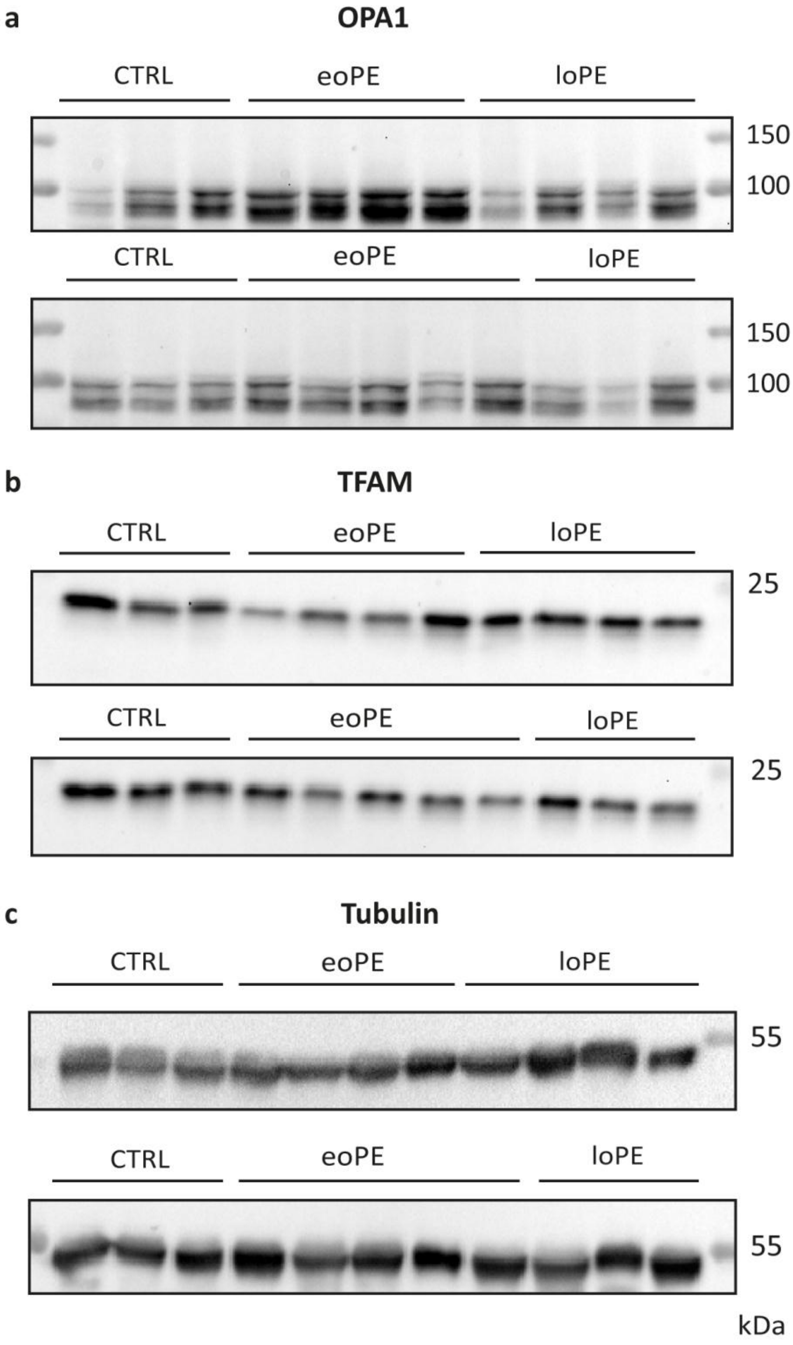

Supplementary Figure S1. Expression of studied proteins in placenta tissue. Anti-OPA1 (a), anti-TFAM (b), anti-tubulin (c) staining of placental homogenates.

SUPPLEMENTAL METHODS

Supplementary Table S2. Primer sequences.

Gene	Primer sequence: sense and antisense	Product size (bp)
ACTB	5\` GAGCGGGAAATCGTGCGTGACATT 3\`	234
	5\` GATGGAGTTGAAGGTAGTTTCGTG 3\`	
MFN1	5\` GATGACCTGGTGTTAGTAGACAG 3\`	212
	5\` GAGGCATCCCAACGATTATTGAG 3\`	
MFN2	5\` GGACTTGCCTCTCTTCTCATTAT 3\`	143
	5\` CTTCCACCATCCATCCTTCTAC 3\`	
OPA1	5\` GTACCGTTAGCCCTGAGACC 3\`	110
	5\` AGCCATGCCTGATGTCACAG 3\`	
TFAM	5\` CTTATAGGGCGGAGTGGCAG 3\`	242
	5\` CAGCTTTTCCTGCGGTGAAT 3\`	
NRF1	5\` GTGGGGGACAGATCGTCTTG 3\`	88
	5\` ATCTGGACCAGGCCATTAGC 3\`	
mtDNA	5\` ATCCCGCACAAGAGTGCTAC 3\`	124
(D-loop)	5\` GGGGAACGTGTGGGCTATTT 3\`	
MT-ND2	5\` TTAAACTCCAGCACCACGACC 3\`	364
	5\` AAGGGGAGATAGGTAGGAGTAGC 3\`	
B2M	5\` AAAAGATGAGTATGCCTGCCG 3\`	150
	5\` TGCAGAGTGTTATATCAGATGGAT 3\`	

Cell culture. Primary cultures were obtained from fetal part of the placental villous tissue by enzymatic treatment. The tissue was washed with PBS, minced and incubated in 0.25% trypsin EDTA solution (HyClone, USA) at 37°C for 20 minutes. The suspension was centrifuged at 200 g and the pellet was resuspended in complete medium DMEM/F12 (PanEco, Russia), containing 10% fetal bovine serum (HyClone, USA), 50 U/ml penicillin, 50 mcg/ml streptomycin, 2 mM L-glutamine (all – PanEco, Russia). After isolation cells were cultured during 72 hours at 37°C and 5% CO_2 and in the first passage were detached with 0.05% trypsin-EDTA, followed by viability and phenotypic studies. Viability of placental cells was evaluated by flow cytometry using propidium iodide (PI). Cells (3×10^5 cells/well) were labeled with 2 μg/mL PI (Live Technologies, P3566) and analyzed by FACSCalibur (Becton Dickinson, USA).

Immunohistochemistry

Immunohistochemistry was performed according to Abcam IHC staining protocol for paraffin sections and fluorescent detection. Briefly, 3 tissue samples from each group were fixed in 10% neutral buffered formalin, embedded in paraffin, sectioned at 5 µm, and mounted on slides. From each samples 5 sections were obtained. Then sections underwent dewax and rehydration, followed by microwave antigen retrieval in Tris/EDTA pH 9.0 for 20 min. Sections were permeabilized twice for 5 min with permeabilization buffer (1% BSA, 0.025% Triton X-100 on PBS), followed by blocking in 2% normal serum with 1% BSA in PBS for 2 hours at RT. Then sections were incubated overnight at 4°C with anti-OPA1 antibodies (ab157457, 1:100, Abcam, USA). As control the sections were subjected to isotype-matched antibody (ab125938, 1:100, Abcam, USA). Following three washes in PBS, the sections were incubated with Alexa Fluor®555-conjugated secondary antibody (ab150062, 1:1,000, Abcam, USA) for 1 h at RT. Stained sections were finally washed in PBS and nuclear staining was performed by Vectashield mounting medium with DAPI (Vectorlab, USA). Slides were analyzed at the same settings using a confocal laser microscope Carl Zeiss LSM700 equipped with 40X objective. 10 separate confocal images from each section were analyzed with ZEN software, where MFI was calculated.

Western blot analysis. Samples were ground to powder in liquid nitrogen, lysed with ice-cold RIPA buffer, sonicated with 3 pulses for 10 seconds each in VibraCells Ultrasonic Processor (Sonics, USA), and incubated at 65°C for 5 minutes. Thereafter 2x loading buffer was added. Samples were stored at -20°C until used and heated 5 min at 65°C before loading. Placental proteins were separated with 10% sodium dodecyl sulfate polyacrylamide gel electrophoresis (SDS-PAGE), and transferred in wet condition to nitrocellulose membrane (Millipore, USA) using CAPS buffer (10 mM CAPS, 10% ethanol, pH=11.0). Membranes were blocked with 5% milk on tris-buffered saline contained 0.1% Tween (TTBS) for 1 h at RT. Blots were incubated with primary (anti-OPA1–ab119685, 1:1000; anti-TFAM–ab155240, 1:2000; anti-Tubulin–

ab18251, 1:10000; anti-VDAC1– ab154856, 1:2000; anti-DRP1–ab150336, 1:1000; all–Abcam, USA) and peroxidase-conjugated secondary antibodies in 5% milk/TTBS for 1 h at RT. Target proteins were visualized by Novex ECL Kit (Invitrogen, USA) in ChemiDoc station (Biorad, USA). Optical density of the protein bands was measured in ImageLab Software.

Mitochondria isolation was performed by method of differential centrifugation. In our study we used the protocol that resulted mostly in isolation of heavy fraction of mitochondria enriched by cytotrophoblast mitochondria[1]. Placental tissue was cut into blocks (1.5 x 1.5 cm) and washed twice in ice-cold physiological saline (0.9% NaCl). Pieces were minced with scissors and washed with isolation medium (10 mM Tris, 0.25 M sucrose, 0.5 mM EDTA, 0.5 mM EGTA, pH=7.5), supplemented with 0.1% BSA. Minced tissue was homogenized with blade-type homogenizer and medium for isolation was added: 5 ml per 1 g of the tissue. All procedures were conducted on ice. Homogenate was centrifuged for 10 min at 4°C, 1,000 g to precipitate heavy fractions, such as nucleus, Golgi apparatus, unbroken cells and tissue fragments. The supernatant was collected and centrifuged for 17 min 7,200 g at 4°C to precipitate mitochondria fraction. The pellet was resuspended in isolation medium without BSA with the same ratio between medium volume and weight of initial tissue. Thereafter, the suspension was centrifuged under the same conditions. Upon centrifugation, the supernatant was discarded and the pellet was resuspended in the minimum volume of isolation medium with EGTA. Mitochondrial protein concentration was measured by biuret method [2].

Measurement of mitochondria activity. Mitochondrial suspension containing 0.6 mg of protein was placed into oxygraph (Hansatech, UK) chamber filled with respiration medium MIR05 (EGTA 0.5 mM, $MgCl_2 \cdot 6H_2O$ 3 mM, K-lactobionate 60 mM, taurine 20 mM, KH_2PO_4 10 mM, HEPES 20 mM, sucrose 110 mM, BSA 1 g/l) at 25°C [3]. Two protocols were performed to estimate respiration supported by I and/or II complexes substrates. Routine respiration was defined without substrate addition. Non-stimulated respiration was measured in presence of complex I substrates (glutamate 5 mM, malate 1.25 mM). Addition of 50 µM ADP allowed to

measure P/O ratio of oxidative phosphorylation (the ATP produced per oxygen atom reduced by the respiratory chain). To measure maximal rate of coupled respiration (state 3) we used 0.5 mM ADP. Mitochondrial membrane integrity was assessed by availability of externally added cytochrome *c* (0.1 μM) to intermembrane space. Respiration rate in state 4 was measured in presence of ATP-synthase inhibitor oligomycin (2.5 μM). Maximum rate of uncoupled respiration was measured in presence of FCCP with titration until maximum rate was reached (0.2 nmoles – each addition). The second protocol was performed to estimate respiration efficiency in presence of 1 μM rotenone and 5 mM succinate. All other additions were done as described above in the first protocol. Oxygen consumption rates were normalized to mitochondrial protein concentration measured by biuret method and expressed as $nmol*min^{-1}*mg^{-1}$ of protein.

Determination of mitochondrial membrane potential (*Δψ*) and sensitivity of mitochondria to Ca^{2+} exposure. Mitochondrial *Δψ* measurement was performed in mitochondrial suspension through fluorescence changes of lipophilic cationic dye safranin O (final concentration 4.3 μM) at 485/586 nm excitation/emission wavelengths, recorded with Cary Eclipse fluorescence spectrophotometer (Agilent Technologies, USA). Mitochondrial suspension containing 1.0 mg of protein was placed into cuvette containing 2 ml Standard reaction medium (250 mM sucrose, 10 mM HEPES buffer, 200 μM EGTA, 2 mM H_2PO_4, 1 mM $MgCl_2$, pH 7.2) with complex I-linked substrate cocktail (malate 1.25 mM, 2.0 mM pyruvate, 5.0 mM glutamate), at 25°C. Rate of Ca^{2+}-induced mitochondrial membrane depolarization was estimated using stepwise titration with 1M $CaCl_2$ (up to 135 μM final concentration in cuvette). Mitochondrial *Δψ* gradual dissipation was assessed with stepwise additions of 10^{-5} M FCCP up to 25 nM in cuvette. Data were calibrated using K^+ gradient as described by Figueira and colleagues [4]. A calibration curve was fitted using Prism software (GraphPad, USA), and all recorded fluorescence tracks were converted into *Δψ* according to the fitting equation.

REFERENCES

1. Martínez, F., Kiriakidou, M. & Strauss, J. F. Structural and functional changes in mitochondria associated with trophoblast differentiation: methods to isolate enriched preparations of syncytiotrophoblast mitochondria. *Endocrinology* **138,** 2172–83 (1997).
2. Gornall, A. G., Bardawilll, C. J. & David, M. M. DETERMINATION OF SERUM PROTEINS BY MEANS OF THE BIURET REACTION. *J. Biol. Chem.* **177,** 751–766 (1949).
3. Gnaiger, E. *et al.* in *Life in the Cold* (eds. Heldmaier, G. & Klingenspor, M.) 431–442 (Springer Berlin Heidelberg, 2000). doi:10.1007/978-3-662-04162-8_45
4. Figueira, T. R., Melo, D. R., Vercesi, A. E. & Castilho, R. F. Mitochondrial Bioenergetics. *Mitochondrial Bioenerg. Methods Protoc. (Methods Mol. Biol. vol. 810)* **810,** 103–117 (2012).
